# Renoprotective Mechanism of Remote Ischemic Preconditioning Based on Transcriptomic Analysis in a Porcine Renal Ischemia Reperfusion Injury Model

**DOI:** 10.1371/journal.pone.0141099

**Published:** 2015-10-21

**Authors:** Young Eun Yoon, Kyung Hwa Choi, Sook Young Kim, Young In Cho, Kwang Suk Lee, Kwang Hyun Kim, Seung Choul Yang, Woong Kyu Han

**Affiliations:** 1 Department of Urology, Urological Science Institute, Yonsei University College of Medicine, Seoul, Korea; 2 Department of Urology, CHA Bundang Medical Center, CHA University, Seongnam-si, Korea; 3 Brain Korea 21 PLUS Project for Medical Science, Yonsei University, Seoul, Korea; 4 Department of Urology, Ewha Women’s University Mokdong Hospital, Seoul, Korea; University of Colorado Denver, UNITED STATES

## Abstract

Ischemic preconditioning (IPC) is a well-known phenomenon in which tissues are exposed to a brief period of ischemia prior to a longer ischemic event. This technique produces tissue tolerance to ischemia reperfusion injury (IRI). Currently, IPC’s mechanism of action is poorly understood. Using a porcine single kidney model, we performed remote IPC with renal IRI and evaluated the IPC mechanism of action. Following left nephrectomy, 15 female Yorkshire pigs were divided into three groups: no IPC and 90 minutes of warm ischemia (control), remote IPC immediately followed by 90 minutes of warm ischemia (rIPCe), and remote IPC with 90 minutes of warm ischemia performed 24 hours later (rIPCl). Differential gene expression analysis was performed using a porcine-specific microarray. The microarray analysis of porcine renal tissues identified 1,053 differentially expressed probes in preconditioned pigs. Among these, 179 genes had altered expression in both the rIPCe and rIPCl groups. The genes were largely related to oxidation reduction, apoptosis, and inflammatory response. In the rIPCl group, an additional 848 genes had altered expression levels. These genes were primarily related to immune response and inflammation, including those coding for cytokines and cytokine receptors and those that play roles in the complement system and coagulation cascade. In the complement system, the membrane attack complex was determined to be sublytic, because it colocalized with phosphorylated extracellular signal-regulated kinase. Furthermore, alpha 2 macroglobulin, tissue plasminogen activator, uterine plasmin trypsin inhibitor, and arginase-1 mRNA levels were elevated in the rIPCl group. These findings indicate that remote IPC produces renoprotective effects through multiple mechanisms, and these effects develop over a long timeframe rather than immediately following IPC.

## Introduction

Ischemic preconditioning (IPC) is an effective method for protecting organs prior to ischemia reperfusion injury (IRI). Since the initial report of the IPC phenomenon, numerous studies have tried to incorporate IPC to protect various organs, including the heart, lung, small intestine, liver, brain, and kidney [1–6]. There are 2 types of IPC. Local IPC involves brief interruption of blood supply to the target organ alternated with brief episodes of reperfusion applied prior to prolonged lethal ischemia. In remote IPC, intermittent brief ischemia and reperfusion is applied to an organ (e.g., a limb or the intestine) distant to the target organ. For organs that are vulnerable to short-term ischemic injury, such as the brain, heart, or kidney, remote IPC is the preferred method [7–9].

Many studies have demonstrated renoprotective effects for remote IPC in renal IRI using rodent animal models [10]. Remote IPC can decrease blood urea nitrogen, creatinine, and malondialdehyde levels [11, 12]. In addition to these biomarkers, histological evidence supporting the renoprotective effects of remote IPC has been reported [13]. While encouraging, these results do not directly translate to the clinical application of remote IPC in humans, because human renal physiology is different from that of rodents [14]. While a few studies have investigated remote IPC in large animals [10, 15, 16], most large animal studies focused on local IPC [17–22]. To safely use IPC in human renal IRI, we first need to determine the most effective IPC protocol and understand its exact mechanism of action. Previous studies have not defined a mechanism of action for remote IPC. Several potential mechanisms have been suggested, including production of adenosine, bradykinins, opioids, antioxidants, humoral substances, and inflammatory molecules; however, there are no accurate and comprehensive reports regarding the remote IPC mechanism of action [23–27]. Recently, gene microarray technology has been used to analyze the molecular basis of many diseases. For example, genetic changes produced by renal IRI have been analyzed using microarrays [28]. However, microarray analysis of remote IPC tissues is limited, and, to our knowledge, there has been no analysis of the transcriptome of renal tissue after remote IPC [29, 30].

In our previous study, we concluded that remote IPC elicited fewer renal injury biomarkers following IRI; however, more animal studies deciphering the detailed remote IPC mechanism are needed before IPC can be incorporated into human surgeries [31]. In this study, we used a microarray analysis to evaluate the effect of the time between IPC and IRI in remote IPC and identify the exact mechanism of action for remote IPC in renal IRI using a porcine single kidney model.

## Materials and Methods

### Animals and design of experiments

**Fig 1:**
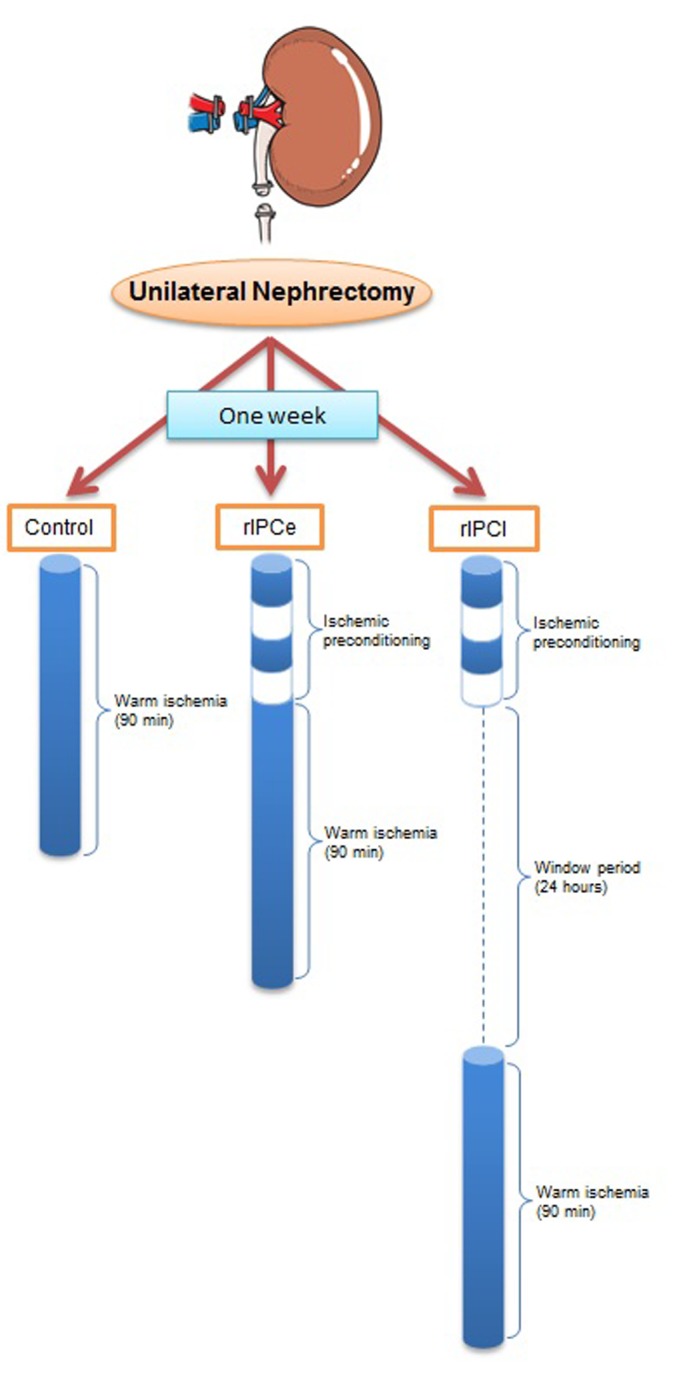
Experimental protocol. Blue cylinders represent the time zone of clamping and clear cylinders represent the declamping. Control, no IPC and 90 minutes of warm ischemia; rIPCe, 40 minutes of remote IPC immediately followed by 90 minutes of warm ischemia; and rIPCl, 40 minutes of remote IPC with 90 minutes of warm ischemia 24 hours later.

The study was approved by the Institutional Animal Care and Use Committee, Yonsei University Health System in accordance with the ‘Guide for the Care and Use of Laboratory Animals’ published by the National Institutes of Health and was conducted according to the principles of the Declaration of Helsinki. A total of 15 female Yorkshire pigs (20 weeks old; 35–38 kg, *S*. *s*. *domesticus*) were used (XP Bio Inc., Anseong-si, Korea). The animals were individually caged for 10 days before the start of the experiment and received standard laboratory food with free access to water. In a prospective design, pigs were allocated randomly to three experimental groups (Fig 1). Group 1 pigs (control, n = 5) underwent right renal ischemia without IPC. Group 2 pigs (rIPCe, n = 5) underwent remote IPC just before right renal ischemia, i.e., an early time window. Group 3 pigs (rIPCl, n = 5) underwent remote IPC with right renal ischemia 24 hours later, i.e., a late time window. All three groups underwent the same protocol for preoperative care, anesthesia, postoperative care, and sacrifice. Prior to anesthesia administration, the pigs were starved for 8 hours. Anesthesia was induced by intramuscular tiletamine-zolazepam (5 mg/kg) and xylazine (2 mg/kg). Following endotracheal intubation, anesthesia was maintained using controlled ventilation with 2% isoflurane at a respiratory rate of 15/min. All procedures were performed laparoscopically with standard 5-mm 3-port access by two surgeons (W.K.H. and Y.E.Y.). During the procedure, intraperitoneal pressure was maintained at 12 mmHg. To create the single kidney porcine model, we first performed a left nephrectomy. After a 1-week renal compensation period, we performed the IPCs and right renal ischemias, as appropriate, based upon the group assignment. We performed two cycles of IPC by clamping the right external iliac artery for 10 minutes followed by 10 minutes of reperfusion. We performed warm ischemia for the right kidney by clamping the right renal artery and vein together for 90 minutes. Antibiotic prophylaxis was administered using amoxicillin clavulanate (15 mg/kg/day), and meloxicam (0.2 mg/kg/day) was used as postoperative analgesia. Pigs were individually housed and had free access to water and feed. Every pig was sacrificed 72 hours postoperatively. Pigs were administered an intramuscular injection of ketamine (25 mg/kg) and xylazine (2 mg/kg) followed by an intravenous potassium chloride injection. Renal tissues were harvested immediately and stored in a -80°C freezer until the microarray analysis was performed.

### Microarray analysis

Total RNA was extracted from tissues using TRIzol (Invitrogen Life Technologies, Carlsbad, CA, USA) according to the manufacturer's protocol and purified with the RNeasy Mini Kit (Qiagen Inc., Valencia, CA, USA). RNA labeling and hybridization were performed using the Agilent One-Color Microarray-Based Gene Expression Analysis protocol (Agilent Technologies, v6.5, Palo Alto, CA, USA). Briefly, 200 ng of total RNA from each sample was linearly amplified and labeled with Cy3-dCTP. The labeled cRNAs were hybridized onto the Porcine Gene Expression Microarray, 4×44K (Agilent Technologies). The hybridized array was immediately scanned with an Agilent Microarray Scanner (Agilent Technologies). Raw data were extracted using the Agilent Feature Extraction Software (Agilent Technologies, v11.0.1.1). The selected gProcessedSignal value was transformed logarithmically and normalized using the quantile method. The expression data were quantified as fold change, and the statistical significance was determined using an independent t-test. We adjusted the p-value to account for the false discovery rate using the Benjamini-Hochberg algorithm. A K-means clustering analysis was performed using the MacQueen algorithm to compare the expression profiles. Hierarchical cluster analysis was performed using complete linkage and Euclidean distance as a measure of similarity. Gene-enrichment and functional annotation analysis for significant probes was performed using DAVID (Database for Annotation, Visualization and Integrated Discovery v6.7, National Institute of Allergy and Infectious Diseases, Bethesda, MD, USA; http://david.abcc.ncifcrf.gov). All data analysis and differentially expressed gene visualization was performed using the R statistic software (R version 3.0.1, R Foundation for Statistical Computing, Vienna, Austria; http://www.r-project.org).

### Immunohistological staining and microscopic analysis

Kidneys were embedded in paraffin, and 2-μm sections were prepared for fluorescent immunostaining and immunohistochemical staining. Deparaffinized renal sections were fixed and incubated in PBS with 5% bovine serum albumin and 0.05% Tween 20 (PBST) at room temperature for 1 h in a humidified chamber to block non-specific binding sites. The sections were incubated overnight with the primary antibodies in PBST buffer with 3% BSA at 4°C. The primary antibodies used were anti-C3c-FITC (Abcam, Cambridge, MA, USA), mouse anti-membrane attack complex (MAC; Abcam), and rabbit anti-phosphorylated extracellular signal-regulated kinase (ERK) 1 and ERK2 (P-ERK; Cell Signaling Technology Inc., Danvers, MA, USA). After washing, the sections were incubated with fluorophore-conjugated secondary antibodies (anti-rabbit Alexa Fluor 488 and anti-mouse Alexa Fluor 594, Invitrogen Life Technologies) for 1 h at room temperature. The sections were mounted using Vectashield mounting medium with 4ʹ,6-diamidino-2-phenylindole (Vector Laboratories, Burlingame, CA, USA). For immunohistochemical staining, the slides were pretreated in 0.01 M citric acid buffer (pH 6.0) for 10 minutes. After blocking, sections were incubated with primary antibody against P-ERK at 4°C overnight. After washing, sections were exposed to a streptavidin-horseradish-peroxidase complex for 30 minutes at 37°C and visualized with 3,3ʹ-diaminobenzidine.

### Terminal Deoxynucleotidyl Transferase-mediated dUTP-X Nick End Labeling (TUNEL) staining

To detect apoptotic cells, TUNEL staining was performed using a TACS^®^ 2 TdT-DAB *In Situ* Apoptosis Detection Kit (Trevigen, Inc., Gaithersburg, MD, USA) according to the manufacturer’s instructions. Kidneys were embedded in paraffin, and 2-μm sections were prepared for TUNEL staining. After deparaffinization, digestion with proteinase K (15 minutes), and quenching of endogenous peroxidase in 2% H_2_O_2_ (5 minutes), formalin-fixed renal sections were immersed in the TdT buffer and incubated with TdT, 1 mM Mn^2+^, and biotinylated dNTP at 37°C for 60 minutes. Then the sections were incubated with streptavidin–horseradish peroxidase at room temperature for 10 minutes and immersed in diaminobenzidine substrate for 5 minutes. The slides were then counterstained with 1% methyl green for 30 seconds.

### Reverse transcriptase-polymerase chain reaction

Microarray results were validated using RT-PCR. Briefly, cDNA was synthesized from purified RNA using oligo dT-primers with SuperScript^TM^ II reverse transcriptase (Invitrogen Life Technologies) according to the supplier’s protocol. Primer sequences used in the study are listed in S1 Table. Equal volumes of amplified cDNAs were loaded into 1.5% agarose gels and separated by electrophoresis. The bands were identified using a Bio-Rad Gel Doc 2000 system with Bio-Rad TDS Quantity One Software.

## Results

### Microarray

All RNA samples had 260/280 ratios between 1.8–2.2, and agarose gel electrophoresis showed two clear bands for 28S and 18S rRNA. In the microarray, 43,603 probes were detected. Among those, 1,053 probes had significant intensity changes. The criteria for significance was |fold change| ≥2 and a p-value <0.05.

**Fig 2:**
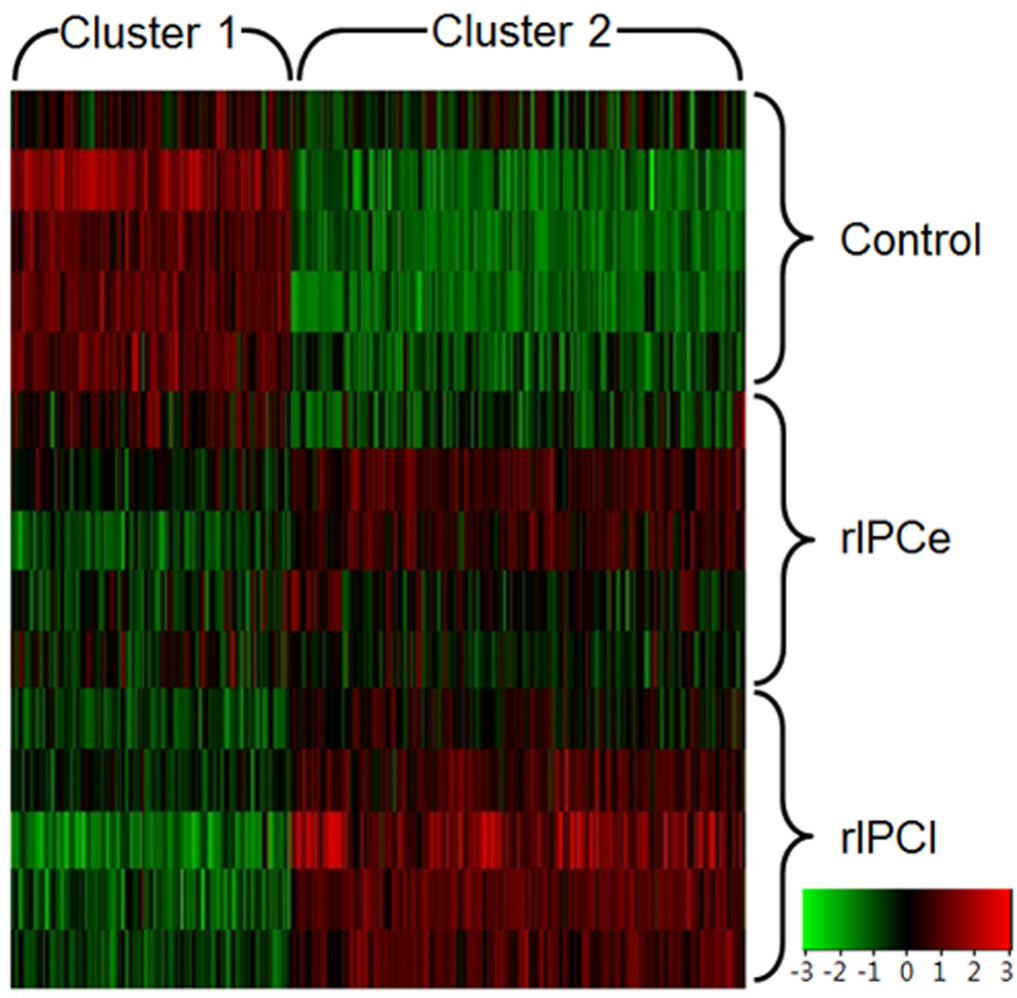
Hierarchal cluster analysis of 1,053 altered genes. The genes were divided into two clusters via K-mean hierarchal cluster analysis. Cluster 1 genes are downregulated (green) in the rIPCe and rIPCl. Cluster 2 genes are upregulated in rIPCl. rIPCe, remote ischemic preconditioning immediately prior to ischemia (early); rIPCl, remote ischemic preconditioning followed by ischemia 24 hours later (late).

The K-mean hierarchal cluster analysis divided all 1,053 genes into two clusters (Fig 2). Cluster 1 consisted of 400 genes that were downregulated in rIPCe relative to the control group and downregulated further in rIPCl. Cluster 2 consisted of 653 genes that were upregulated in rIPCe relative to the control group and upregulated further in rIPCl.

**Fig 3:**
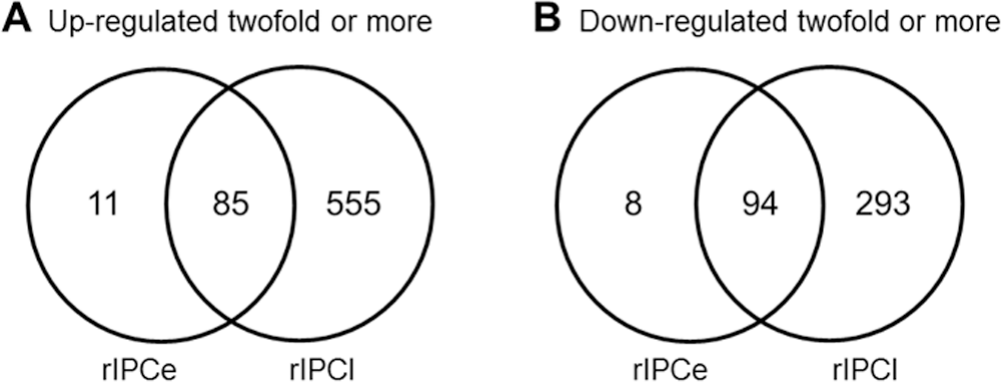
Venn diagrams of altered gene expression. rIPCe, remote ischemic preconditioning immediately prior to ischemia (early); rIPCl, remote ischemic preconditioning followed by ischemia 24 hours later (late).

The rIPCe group had 198 genes with expression level changes; 96 genes were upregulated, and 102 genes were downregulated compared to those in the control group (Fig 3). The rIPCl group had 1,027 genes with expression level changes; 640 genes were upregulated, and 387 genes were downregulated. Fig 3 shows that nearly all genes with altered expression in the rIPCe group were also differentially expressed in the rIPCl group.

### Altered gene expression shared by rIPCe and rIPCl

**Fig 4:**
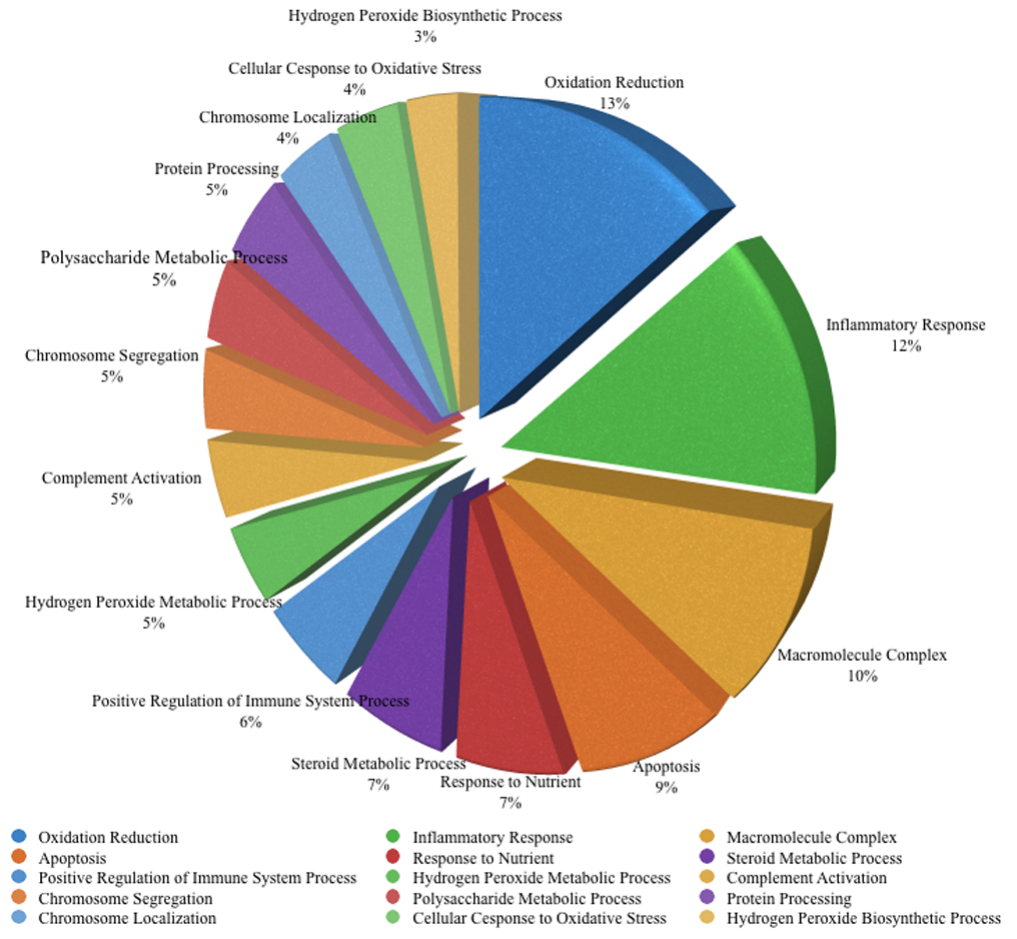
Major enriched gene ontology biological processes for the 179 genes with similar expression levels in the rIPCe and rIPCl groups. Similar gene ontology terms were excluded to avoid repetition. rIPCe, remote ischemic preconditioning immediately prior to ischemia (early); rIPCl, remote ischemic preconditioning 24 hours followed by ischemia 24 hours later (late).

The rIPCe and rIPCl groups had 179 genes with similar expression levels; 85 genes were upregulated and 94 genes were downregulated compared to the control group genes. The similarly expressed genes were primarily related to oxidation, inflammation, macromolecule complex assembly, and apoptosis (S2 Table). Gene ontology enrichment analysis confirmed that some of the genes were related to oxidative reduction, inflammatory response, macromolecular complex assembly, and apoptosis. Gene ontology analysis also identified additional functions (Fig 4); however, these genes were not part of a specific pathway.

### Altered gene expression in the rIPCl group

**Fig 5:**
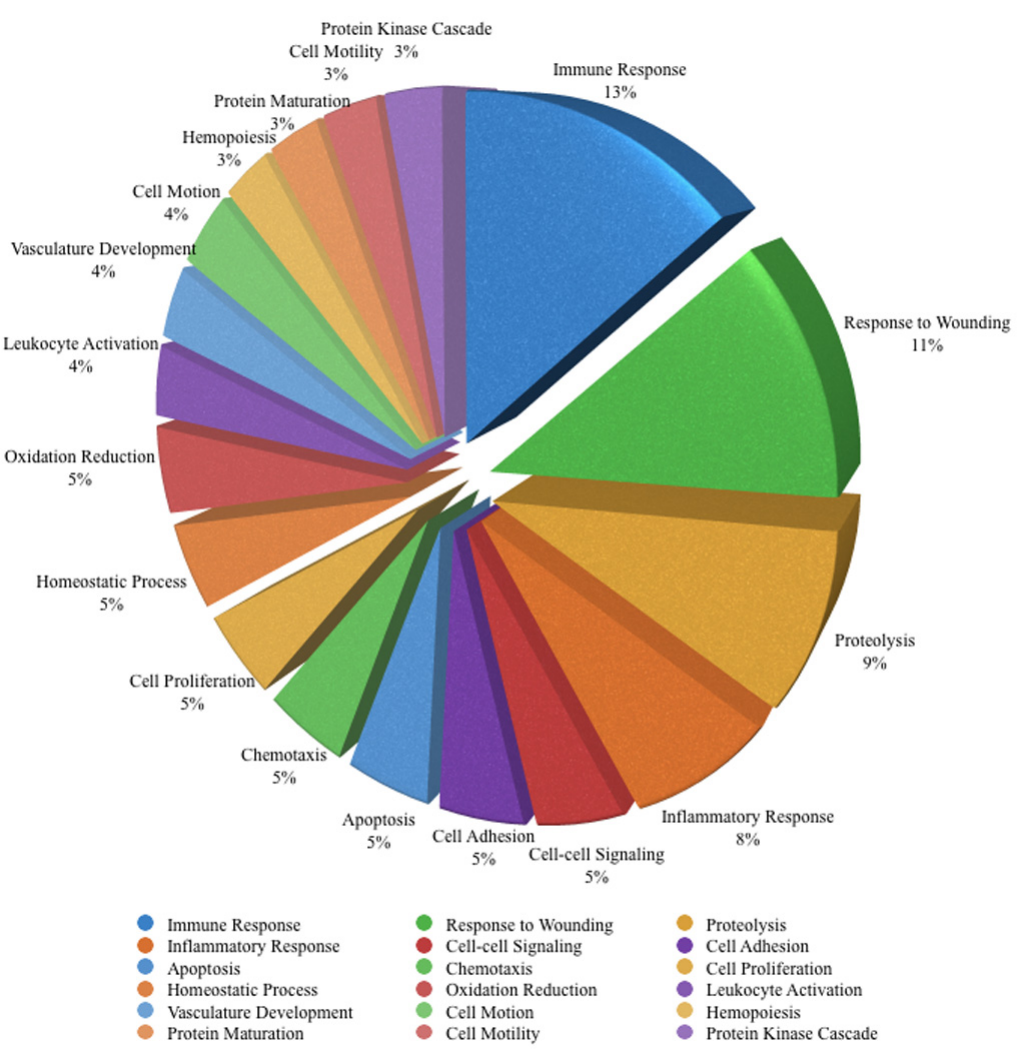
Major enriched gene ontology biological processes for the 848 genes with altered expression levels in the rIPCl group only. Similar gene ontology terms were excluded to avoid repetition. rIPCl, remote ischemic preconditioning followed by ischemia 24 hours later (late).

Among the 1,053 genes in the rIPCl group with altered expression levels compared to the control group, 848 genes were altered in the rIPCl group only. Of those, 555 genes were upregulated, and 293 genes were downregulated. Gene ontology enrichment analysis showed that the rIPCl group genes with altered expression levels were related to immune response, response to wounding, proteolysis, and inflammation rather than apoptosis or oxidation (Fig 5).

### Functional classification of genes

**Fig 6:**
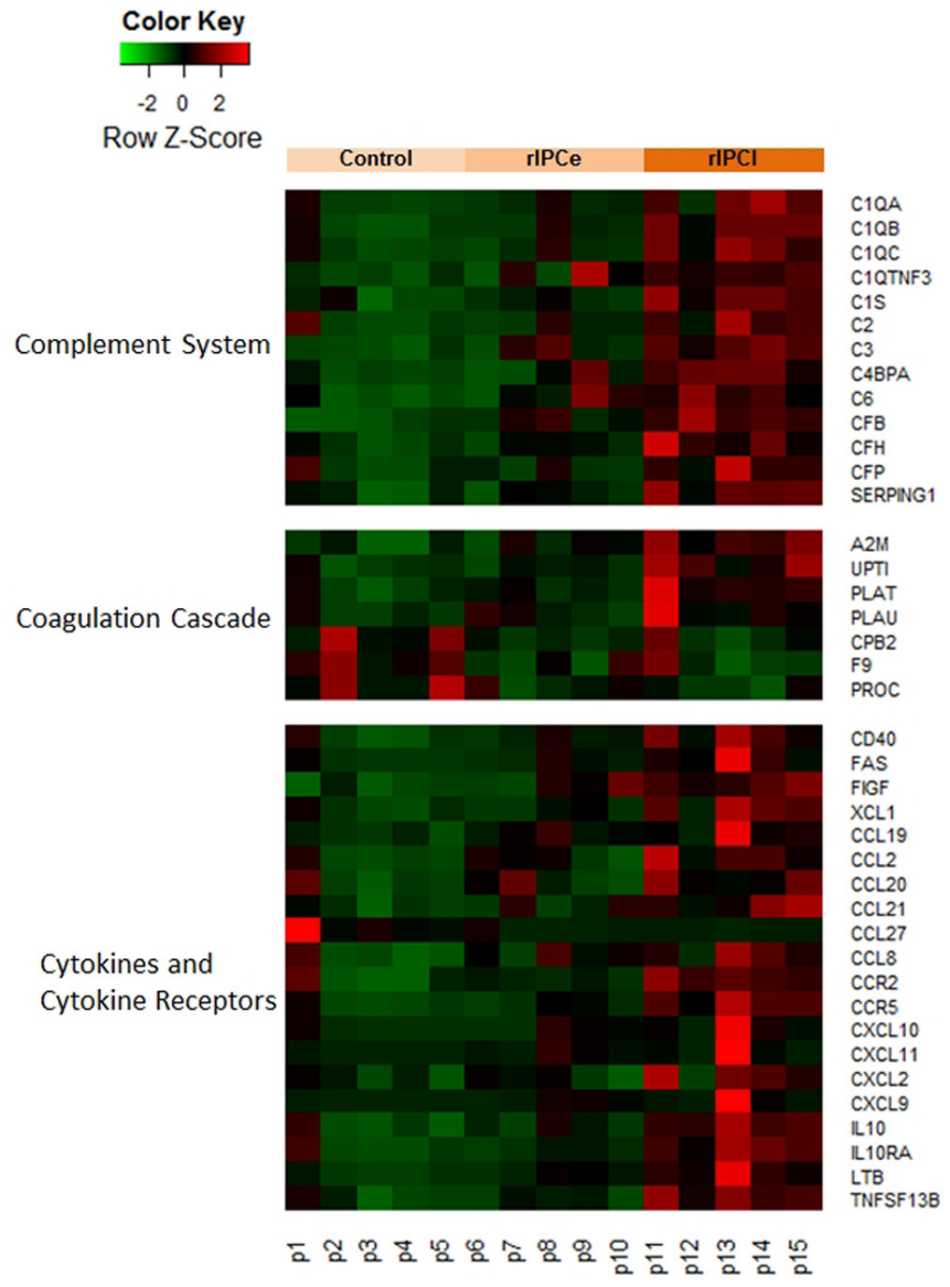
Heatmap signature of suggested pathways by DAVID. rIPCe, remote ischemic preconditioning immediately prior to ischemia (early); rIPCl, remote ischemic preconditioning followed by ischemia 24 hours later (late).

We attempted to identify some potential IPC mechanisms with DAVID. When we examined the entire list of altered expression level genes, DAVID noted several enriched pathways (S3 Table), including the complement system, the coagulation cascade, and cytokines and cytokine receptors (Fig 6). The heatmap shows that most of these genes were upregulated in the rIPCl group (Fig 6).

### The complement system

**Fig 7:**
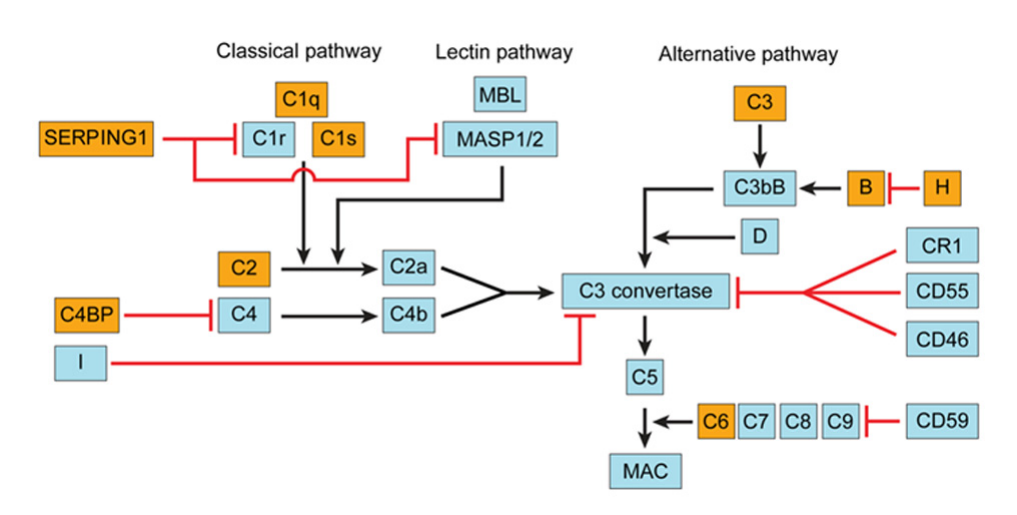
Genes in the complement system with altered expression levels. The yellow boxes indicate upregulated gene expression (fold change ≥2 and p < 0.05). The red lines indicate inhibition.

We detected expression changes in many complement cascade genes in the rIPCl group compared to the control group (Fig 7). The classical pathway genes C1q, C1s, and C2 were upregulated. The alternative pathway genes C3 and factor B were also upregulated. However, complement inhibitors, including serpin peptidase inhibitor, clade G (C1 inhibitor), member 1 (*SERPING1*), C4b-binding protein (*C4BP*), and factor H, were also upregulated.

**Fig 8:**
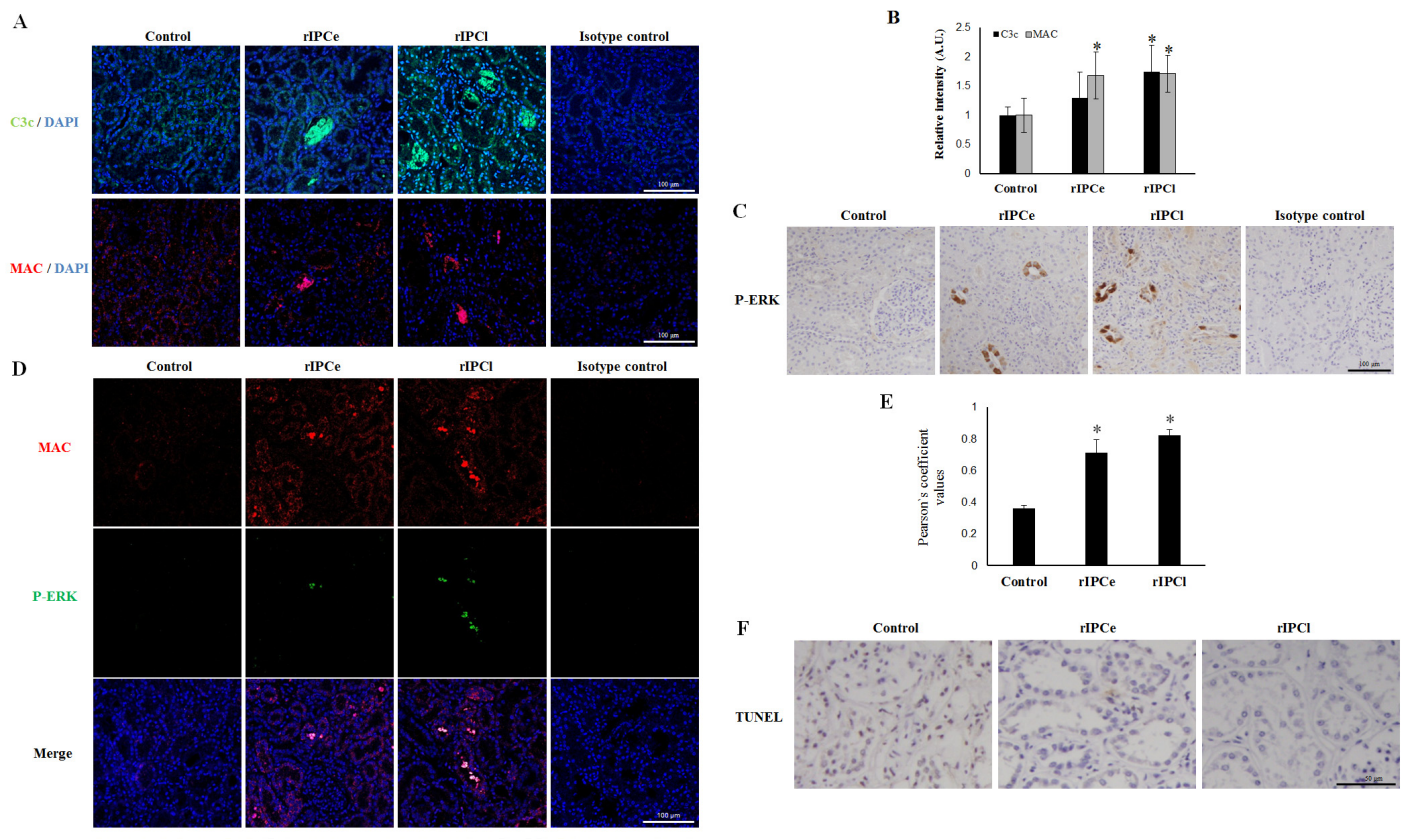
Immunohistochemical analysis of pig renal tissue. (A) C3c (green) and MAC (red) deposition was observed in confocal images of renal tissues, scale bar = 100 μm; DAPI, 4ʹ,6-diamidino-2-phenylindole (blue). (B) Relative fluorescence intensity of C3c and MAC staining was increased in rIPCe and rIPCl, *p < 0.05 vs. Control. (C) P-ERK IHC shows that ERK is activated in rIPCe and rIPCl, scale bar = 100 μm. (D) MAC (red) and P-ERK (green) colocalization was observed in confocal images of renal tissues, scale bar = 100 μm. (E) Pearson's coefficient for MAC and P-ERK colocalization (in 12 fields) was higher in rIPCe and rIPCl, *p < 0.05 vs. Control. (F) TUNEL staining showed fewer apoptotic renal tubular cells in the rIPCl group compared with Control, scale bar = 50 μm. MAC, membrane attack complex; P-ERK, phosphorylated extracellular signal-regulated kinase; rIPCe, remote ischemic preconditioning immediately prior to ischemia (early); rIPCl, remote ischemic preconditioning followed by ischemia 24 hours later (late); TUNEL, terminal deoxynucleotidyl transferase dUTP nick end labeling.

To confirm complement pathway activation, we performed immunofluorescent staining for C3c, a cleavage product of activated C3 and MAC, using a monoclonal antibody against neoantigen (Fig 8A). C3c and MAC deposition was observed in both the rIPCe and rIPCl groups. The relative fluorescence intensity of C3c and MAC was higher in the rIPCl group than that in the rIPCe group (Fig 8B). Because ERK is activated by sublytic MAC formation, we evaluated P-ERK levels in the renal tissues. The rIPCl group renal tubular cells were strongly positive for P-ERK staining, whereas control group renal tubular cells showed no staining (Fig 8C). MAC and P-ERK colocalization was also detected in the rIPCl group (Fig 8D), and the Pearson's coefficient for MAC and P-ERK colocalization in 12 fields was higher in the rIPCl group than in the control and rIPCe groups (Fig 8E). TUNEL staining revealed that the rIPCl group had fewer apoptotic cells in renal tubules compared with those in the control or rIPCe groups (Fig 8F).

### The coagulation cascade

**Fig 9:**
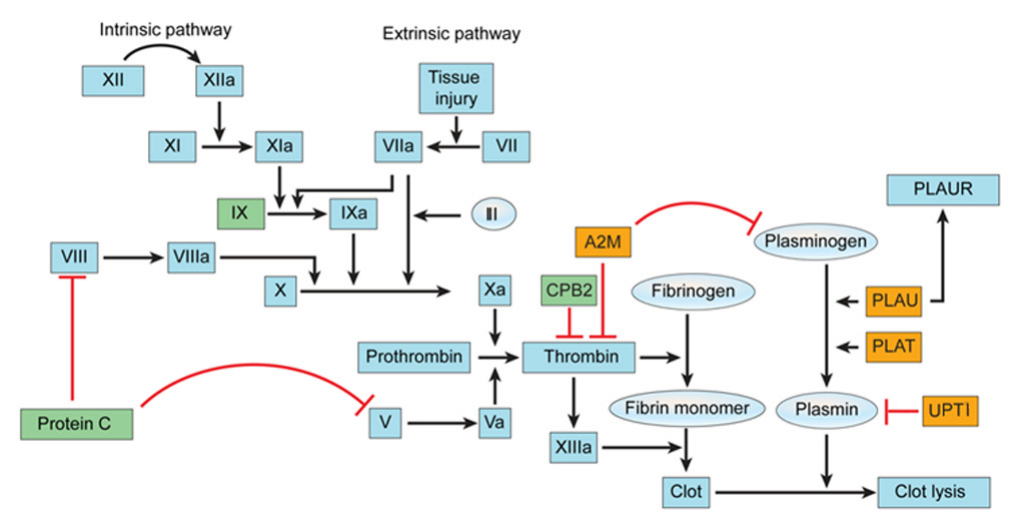
Genes in the coagulation cascade with altered expression levels. Yellow boxes indicate upregulated genes (fold change ≥2 and p < 0.05). Green boxes indicate downregulated genes (fold change ≤-2 and p < 0.05). Red lines indicate inhibition.

Many of the differentially expressed genes in the rIPCl group were involved in the coagulation cascade (Fig 9). Plasma thromboplastin antecedent (IX), protein C, and carboxypeptidase B2 (*CPB2*) were downregulated in the rIPCl group. In contrast, alpha 2 macroglobulin (*A2M*), tissue plasminogen activator (*PLAT*), and urokinase-type plasminogen activator (*PLAU*) were upregulated in the rIPCl group.

We used RT-PCR analysis to validate the gene expression changes detected during the microarray analysis. The mRNA levels of *A2M*, *PLAT*, and uterine plasmin trypsin inhibitor (*UPTI*) were elevated in both the rIPCe and rIPCl groups when compared with those in the control group (S1 Fig). While the *A2M* mRNA level in the rIPCl group was not upregulated to the same degree as that in the rIPCe group, the *A2M* expression levels in both groups were markedly elevated compared to that of the control group. These results were consistent with the microarray data. However, the RT-PCR analysis did not demonstrate any changes in the *PLAU* expression level in either the rIPCe or rIPCl groups, which contradicted the microarray data indicating that *PLAU* expression was upregulated in the rIPCl group (S1 Fig).

### Cytokines and cytokine receptors

S4 Table lists the cytokines and cytokine receptors that the microarray analysis identified as differentially expressed in the rIPCe and rIPCl groups. Most of the cytokines and cytokine receptors were upregulated in the rIPCl group compared to those in the control group. We performed RT-PCR to validate the expression levels of interleukin 10 (IL-10) and transforming growth factor beta 1 (TGF-β1). The *IL-10* and *TGF-β1* mRNA levels were upregulated in both the rIPCe and rIPCl groups compared to those in the control group, confirming the microarray results (S1 Fig and S4 Table). The RT-PCR also verified that the M2 mononuclear phagocyte marker, arginase-1 (*ARG-1*) mRNA expression level was upregulated in both the rIPCe and rIPCl groups (S1 Fig).

## Discussion

Renal IRI, which is caused by cardiopulmonary bypass, partial nephrectomy, and renal transplantation, is an emerging clinical problem, because acute renal injury can result in end-stage renal disease [32]. Chronic kidney disease increases the risk of death and cardiovascular morbidity, even when dialysis is unnecessary [33]. With this in mind, IPC may be an alternative strategy to preserve renal function after IRI.

In our previous study, we demonstrated that remote IPC with the late window group had lower urinary kidney injury molecule-1 (KIM-1) and neutrophil gelatinase-associated lipocalin (NGAL) levels than the control group following IRI [31]. Gardner *et al*. also demonstrated that remote IPC has a renoprotective effect in renal IRI in a porcine model [16]. Our results demonstrate that numerous gene expression levels were altered in the rIPC groups, especially in the rIPCl group. Hanto et al. verified that intraoperative administration of inhaled carbon monoxide reduces delayed graft function in a porcine delayed kidney transplantation model [34]. In that study, the authors hypothesized that the expression of antioxidant, proinflammatory, and reparative gene families improved renal function. Jun et al. performed a microarray study demonstrating a protective effect for IPC in a lung IRI model [29]. In their study, the differentially expressed genes had roles in inflammation, apoptosis, oxidation, antioxidation, and metabolism. While their study evaluated lung tissue rather than renal tissue, the microarray analysis results are similar to those from our present study, which provides additional evidence for our hypothesis that IPC affects numerous pathways rather than one specific pathway. Our microarray data suggest that IPC induces changes in the complement system, the coagulation cascade, and various cytokines and cytokine receptors.

### The complement system

Of the three major complement pathways, the alternative and classical pathways are active in the kidney [35, 36]. A previous study confirmed that the complement system, especially the C3 protein, plays a major role in tissue damage in IRI [37]. In our study, the classical pathway proteins C1s, C1q, and C2 and the alternative pathway protein C3 were upregulated in the rIPCl group. However, inhibitors of these proteins (*SERPING1*, *C4BP*, and factor H) were also upregulated. In a kidney transplantation model, Castellano et al. demonstrated that C1 inhibitor infusion significantly reduced tubular damage and tubular epithelial cell apoptosis [38]. These authors concluded that classical and lectin pathway inhibition may provide an alternative therapeutic approach for renal IRI. Other studies demonstrated that a mutation in the factor H gene, a regulator of the alternative pathway, is a risk factor for some renal diseases, which supports the idea that complement inhibition has beneficial effect in IRI [39]. Factor H also has a major role in renal IRI, and a recombinant form of factor H reduces complement activation in the tubulointerstitium after renal IRI [40].

Our microarray data demonstrated upregulation of various complement cascade proteins and IRI-protective regulatory proteins, such as *SERPING1* and Factor H. These results suggest that IPC may reduce IRI damage by neutralizing the complement cascade. We did detect increased C3c and MAC staining in rIPCe and rIPCl tissues; however, phosphorylated ERK colocalized with MAC. The colocalization data suggest that sublytic concentrations of MAC were present in the tissue [41]. Sublytic MAC concentrations reduce the infarction size in hearts [42]. Furthermore, sublytic MAC concentrations induce proliferation in specific cells, such as human aortic smooth muscle cells [43]. Sublytic doses of MAC desensitize cells and make them resistant to cell lysis through ERK activation [44]. Our TUNEL results demonstrated that there were fewer apoptotic cells in the rIPCl group than in the control and rIPCe groups, which agrees with previous studies.

### The coagulation cascade

In our study, we stopped blood flow to produce renal IRI. As a result, coagulation cascade-related genes, such as *PLAT*, *CPB2*, and *A2M*, were differentially expressed in the rIPCl group. These data suggest that IPC may reduce ischemic injury by lysing blood clots. However, these genes may also regulate non-coagulation cascade pathways. Roelofs et al. showed that *PLAT* expression reduces neutrophil influx and preserves renal function during renal IRI using *PLAT* knockout mice [45]. Therefore, *PLAT* may also play an anti-inflammatory role in IPC. *CPB2* mRNA expression was downregulated in the rIPCl group compared with the control group in our study. *CPB2* is a thrombin-activated fibrinolysis inhibitor that eliminates fibrin carboxyl-terminal residues [46]. CPB2 downregulation may be beneficial during IRI, because reduced *CPB2* expression increases fibrinolysis. The microarray analysis also showed that *A2M* expression increased in both the rIPCe and rIPCl groups (2.00- and 2.86-fold changes, respectively), which was verified by RT-PCR. *A2M* inhibits coagulation by inactivating thrombin and inhibits clot lysis by inactivating plasmin [47]. *A2M* also inhibits proteolysis by reducing protease activity [48]. Therefore, *A2M* may reduce ischemic injury following remote IPC by inhibiting coagulation and proteolysis.

### Cytokines and cytokine receptors

CXCL10 and CXCL11 were upregulated in the rIPCe and rIPCl groups in our study. IFN-γ, which is a major macrophage activator, may upregulate the expression of chemokines, such as CXCL9, CXCL10, and CXCL11, in renal proximal tubular epithelial cells [49]. Fibronectin and fibrinogen can then bind to CXCL10 and CXCL11, and the interaction between fibronectin and CXCL11 potentiates wound repair [50]. The anti-inflammatory IL-10 was also upregulated in the rIPCl group. Administering exogenous IL-10 to normal rats stimulates mesangial cell growth, both *in vitro* and *in vivo* [51]. Mesangial cells are critical for maintaining renal function, because they provide structural support to the glomerulus and affect the glomerular filtration rate [52]. In addition, increased IL-10 levels decreased proteinuria and reduced interstitial fibrosis and glomerulosclerosis in a 5/6 nephrectomized rat model [53]. IL-10 also regulates the synthesis of cystatin C, which controls mesangial cell proliferation [54, 55]. Therefore, IL-10 may reduce ischemic injury by promoting mesangial cell proliferation and viability after remote IPC.

TGF-β, another anti-inflammatory cytokine, was upregulated in both rIPC groups compared with the control group. In the kidney, only mesangial cells secrete and activate TGF-β [56]. TGF-β can cause renal epithelial cell hypertrophy [57]. Mesangial cells initiate compensatory renal hypertrophy, even though tubular cells are the major players in the hypertrophic process [58]. Sinuani et al. verified that mesangial cells control the degree of compensatory tubular cell hypertrophy by regulating TGF-β-induced IL-10 expression levels [58]. IL-10 synthesis inhibition in mesangial cells results in a significant TGF-β expression reduction in remnant kidneys. Consequently, there is a 25% reduction in compensatory tubular cell hypertrophy. Taken together, these findings suggest that IPC may contribute to anti-inflammatory effects and tubular cell reproduction by altering IL-10 and TGF-β gene expression. To our knowledge, there are no reports on the roles of IL-10 or TGF-β in relation to the molecular mechanism of remote IPC. Further work is needed to verify the roles of IL-10 and TGF-β in remote IPC and to determine their roles in the kidney.

## Conclusions

These results suggest that remote IPC produces renoprotective effects in renal IRI through multiple mechanisms rather than one specific pathway. Remote IPC produces expression changes in genes related to inflammatory cytokines and cytokine receptors, apoptosis, oxidation and antioxidation, ion channels and aquaporins, metabolism, and the cytoskeleton. Our data suggest that the complement system, the coagulation cascade, and various cytokines and cytokine receptors are the major mechanisms of remote IPC. The changes in these pathways were more pronounced when renal IRI was performed 24 hours after remote IPC rather than immediately afterwards. This study did not determine the optimal time frame to perform remote IPC prior to renal IRI; however, our results provide further evidence that remote IPC is renoprotective for renal IRI. Future studies will focus on elucidating the optimal time window for remote IPC and further defining the functional roles of the various genes identified in this study.

## Supporting Information

S1 Fig RT-PCR analysis of seven genes with altered expression levels according to the microarray data. Glyceraldehyde-3-phosphate dehydrogenase (*GAPDH*) was used as an internal control.
(TIF)

S1 Table Summary of primer sequences used in this study. (DOCX)

S2 Table Representative list of 45 out of the 179 genes with altered expression levels in the rIPCe and rIPCl groups. (DOCX)

S3 Table Pathway signature of IPC identified by DAVID. (DOCX)

S4 Table Cytokines and cytokine receptors with altered gene expression levels. (DOCX)
