# Supplementary figures and images for: Renoprotective Mechanism of Remote Ischemic Preconditioning Based on Transcriptomic Analysis in a Porcine Renal Ischemia Reperfusion Injury Model
Source: PLoS One. 2015 Oct 21;10(10):e0141099. doi: 10.1371/journal.pone.0141099 (PMC4619554; doi:10.1371/journal.pone.0141099)

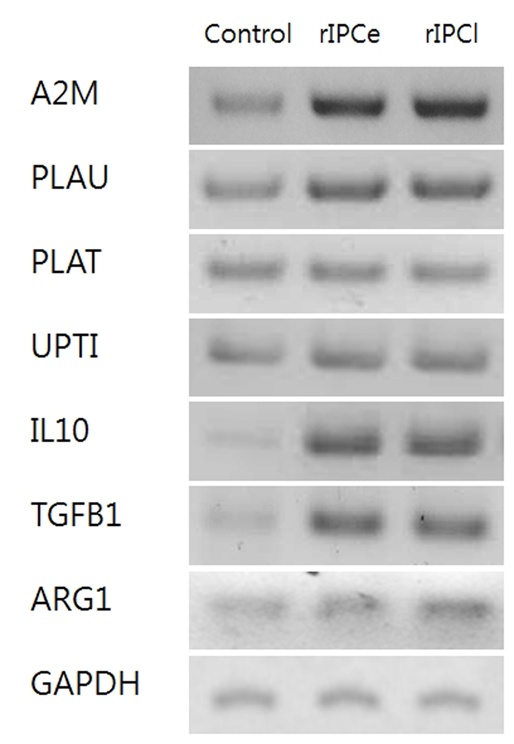

Supplement: S1 Fig — Glyceraldehyde-3-phosphate dehydrogenase (GAPDH) was used as an internal control. (TIF) [file pone.0141099.s001.tif]
