# Supplementary material for: Renoprotective Mechanism of Remote Ischemic Preconditioning Based on Transcriptomic Analysis in a Porcine Renal Ischemia Reperfusion Injury Model
Source: PLoS One. 2015 Oct 21;10(10):e0141099. doi: 10.1371/journal.pone.0141099 (PMC4619554; doi:10.1371/journal.pone.0141099)
Supplement: S1 Table — (DOCX) [file pone.0141099.s002.docx]

**S1 Table. Summary of primer sequences**

| Gene Name | Primer Sequence | |
| --- | --- | --- |
| Alpha 2 macroglobulin | Forward | 5'- TCC ACG GAG AAG AAC TGC TG-3' |
|  | Reverse | 5'- CCA CGT AGT ACA CGA TGG GC -3' |
| Urokinase-type plasminogen activator | Forward | 5'- CTG ACC CAC AGT GGA AAA CG -3' |
|  | Reverse | 5'- GTG GGC TAG GCC ATT CTC TC -3' |
| Tissue plasminogen activator | Forward | 5'- AGG TCC CCA GGA GAG AGG TT -3' |
|  | Reverse | 5'- CAG CCT ATA CGT TCT GCC CA -3' |
| Uterine plasmin trypsin inhibitor | Forward | 5'- TGC CAG AGC TTT GTC TAC GG -3' |
|  | Reverse | 5'- TCA GAA GCA CAG TTA CCC CG -3' |
| Interleukin-10 | Forward | 5'- CTG GAA GAC GTA ATG CCG AA -3' |
|  | Reverse | 5'- GCT CTT GTT TTC ACA GGG CA -3' |
| Transforming growth factor beta-1 | Forward | 5'- TCC ACG GAG AAG AAC TGC TG -3' |
|  | Reverse | 5'- CCA CGT AGT ACA CGA TGG GC -3' |
| Arginase-1 | Forward | 5'- TAC TGG GCG GAG ACC ACA GT -3' |
|  | Reverse | 5'- CCA CCC AAA TGA CAC AGA GAT CT -3' |
| Glyceraldehyde-3-phosphate dehydrogenase | Forward | 5'- GAG AAG TAT GAC AAC TCC CTC AAG A -3' |
|  | Reverse | 5'- CAT CAA AAG TGG AAG AGT GAG TGT C -3' |
