# Supplementary material for: Renoprotective Mechanism of Remote Ischemic Preconditioning Based on Transcriptomic Analysis in a Porcine Renal Ischemia Reperfusion Injury Model
Source: PLoS One. 2015 Oct 21;10(10):e0141099. doi: 10.1371/journal.pone.0141099 (PMC4619554; doi:10.1371/journal.pone.0141099)
Supplement: S2 Table — (DOCX) [file pone.0141099.s003.docx]

**S2 Table.** **Representative list of 45 out of the 179 genes with altered expression levels in the rIPCe and rIPCl groups.**

| Symbol | Gene name | | Ratio* | |
| --- | --- | --- | --- | --- |
|  |  |  | rIPCe | rIPCl |
| **Oxidation Reduction** | | |  |  |
| DUOX1 | | dual oxidase 1 | 2.04 | 2.91 |
| HPD | | 4-hydroxyphenylpyruvate dioxygenase | 0.30 | 0.31 |
| NQO1 | | NAD(P)H dehydrogenase, quinone 1 | 0.17 | 0.11 |
| AKR1CL1 | | aldo-keto reductase family 1, member C-like 1 | 0.40 | 0.27 |
| AKR1C4 | | aldo-keto reductase family 1, member C4 | 0.44 | 0.28 |
| CYP1A1 | | cytochrome P450, family 1, subfamily A, polypeptide 1 | 0.20 | 0.16 |
| CYP24A1 | | cytochrome P450, family 24, subfamily A, polypeptide 1 | 0.34 | 0.35 |
| CYP4F2 | | cytochrome P450, family 4, subfamily F, polypeptide 2 | 0.22 | 0.28 |
| GLUD1 | | glutamate dehydrogenase 1 | 0.42 | 0.31 |
| GPX3 | | glutathione peroxidase 3 | 0.44 | 0.37 |
| GSTA2 | | glutathione S-transferase alpha 2 | 0.15 | 0.22 |
| **Inflammatory response** | | |  |  |
| CLU | | clusterin | 2.79 | 4.03 |
| C4BPA | | complement component 4 binding protein, alpha | 3.54 | 3.72 |
| C6 | | complement component 6 | 2.79 | 2.95 |
| C3 | | complement component 3 | 2.39 | 6.49 |
| FN1 | | fibronectin 1 | 3.54 | 4.77 |
| A2M | | alpha-2-macroglobulin | 2.00 | 2.86 |
| CXCL11 | | chemokine (C-X-C motif) ligand 11 | 4.08 | 8.50 |
| LYZ | | lysozyme | 4.44 | 12.72 |
| CCL27 | | chemokine (C-C motif) ligand 27 | 0.19 | 0.10 |
| ITIH4 | | inter-alpha-trypsin inhibitor heavy chain family, member 4 | 0.47 | 0.37 |
| **Macromolecule complex** | | |  |  |
| APOA4 | | apolipoprotein A-IV | 0.07 | 0.03 |
| APOC3 | | apolipoprotein C-III | 0.24 | 0.25 |
| CENPF | | centromere protein F (mitosin) | 4.36 | 6.70 |
| MGP | | matrix Gla protein | 2.93 | 4.88 |
| KCNA2 | | potassium voltage-gated channel, shaker-related subfamily, member 2 | 0.43 | 0.41 |
| SLC7A9 | | solute carrier family 7 | 0.45 | 0.35 |
| **Apoptosis** | | | | |
| BIRC5 | | baculoviral IAP repeat containing 5 (survivin) | 2.45 | 3.63 |
| CCNB1 | | cyclin B1 | 4.78 | 7.33 |
| CCNB2 | | cyclin B2 | 3.61 | 5.48 |
| PTTG1 | | pituitary tumor-transforming 1 | 4.55 | 6.93 |
| SPAG5 | | sperm associated antigen 5 - astrin ortholog | 2.31 | 5.18 |
| STMN1 | | stathmin 1 | 2.63 | 3.31 |
| DLGAP5 | | discs, large (drosophila) homolog-associated protein 5 | 4.4 | 5.86 |
| **Membrane transport proteins** | | |  |  |
| SLC22A8 | | solute carrier family 22 | 0.47 | 0.42 |
| SLC26A4 | | solute carrier family 26 | 0.25 | 0.14 |
| SLC27A2 | | solute carrier family 27 | 0.44 | 0.34 |
| SLC35D1 | | solute carrier family 35 | 0.43 | 0.22 |
| SLC44A4 | | solute carrier family 44 | 0.46 | 0.33 |
| **Cell adhesion** | |  |  |  |
| COL14A1 | | collagen, type XIV, alpha 1 | 2.00 | 2.52 |
| VCAM1 | | vascular cell adhesion molecule 1 | 2.00 | 2.96 |
| **Others** | |  |  |  |
| LGMN | | legumain | 0.44 | 0.34 |
| SDS | | serine dehydratase | 0.28 | 0.14 |
| GSTO1 | | glutathione S-transferase omega 1 | 0.48 | 0.36 |
| RHOF | | ras homolog family member F | 0.47 | 0.42 |

*Ratio: normalized average signal relative values of the rIPCe group versus the control group and the rIPCl group versus the control group. rIPCe, remote ischemic preconditioning with an early time window; rIPCl, remote ischemic preconditioning with a late time window.
