# Supplementary material for: Renoprotective Mechanism of Remote Ischemic Preconditioning Based on Transcriptomic Analysis in a Porcine Renal Ischemia Reperfusion Injury Model
Source: PLoS One. 2015 Oct 21;10(10):e0141099. doi: 10.1371/journal.pone.0141099 (PMC4619554; doi:10.1371/journal.pone.0141099)
Supplement: S3 Table — (DOCX) [file pone.0141099.s004.docx]

**S3 Table. Pathway signature of IPC identified by DAVID.**

| **Category and Pathway** | | **%** | **P Value** | **Genes** |
| --- | --- | --- | --- | --- |
| **BIOCARTA** | |  |  |  |
|  | Adhesion and Diapedesis of Lymphocytes | 2.1 | < 0.001 | VCAM1, ITGAL, SELL, MADCAM1, ITGB2, ITGA4 |
|  | Cells and Molecules involved in local acute inflammatory response | 2.1 | 0.001 | VCAM1, ITGAL, C3, C6, ITGB2, ITGA4 |
|  | Alternative Complement Pathway | 1.8 | 0.001 | CFP, C3, CFB, C6, C2 |
|  | Complement Pathway | 2.1 | 0.001 | C1QA, C3, CFB, C6, C1S, C2 |
|  | Classical Complement Pathway | 1.8 | 0.002 | C1QA, C3, C6, C1S, C2 |
|  | Adhesion Molecules on Lymphocyte | 1.4 | 0.006 | ITGAL, SELL, ITGB2, ITGA4 |
|  | Monocyte and its Surface Molecules | 1.4 | 0.012 | ITGAL, SELL, ITGB2, ITGA4 |
|  | T Helper Cell Surface Molecules | 1.4 | 0.015 | ITGAL, CD3E, ITGB2, THY1 |
|  | T Cytotoxic Cell Surface Molecules | 1.4 | 0.015 | ITGAL, CD3E, ITGB2, THY1 |
|  | Stathmin and breast cancer resistance to antimicrotubule agents | 1.4 | 0.019 | CCNB1, CDK1, CAMK4, STMN1 |
|  | CTL mediated immune response against target cells | 1.4 | 0.028 | ITGAL, CD3E, ITGB2, FAS |
|  | Neutrophil and Its Surface Molecules | 1.1 | 0.049 | ITGAL, SELL, ITGB2 |
| **REACTOME PATHWAY** | |  |  |  |
|  | Signaling in Immune system | 9.2 | < 0.001 | ITGAL, OLR1, CD3E, SELL, C3, CFB, C6, ICAM3, TLR2, SLC7A9, ITGB2, CD40, C1S, ITGA4, C1QC, TLR9, PROC, VCAM1, C1QA, CD48, C1QB, DOK2, MADCAM1, C2, FN1, TYROBP |
|  | Hemostasis | 7.1 | < 0.001 | PLAT, ITGAL, A2M, OLR1, SELL, CLU, F9, SLC7A9, SERPING1, ITGB2, ITGA4, SOD1, PROC, TIMP1, CD48, DOK2, FIGF, PLAU, SYK, FN1 |
|  | Integrin cell surface interactions | 2.8 | 0.020 | VCAM1, ITGAL, ICAM3, MADCAM1, ITGB2, VTN, ITGA4, FN1 |
|  | Biological oxidations | 3.2 | 0.045 | GSTA2, CYP24A1, CYP1A1, SLC35D1, FMO1, MAOA, CYP2R1, GSTO1, CYP4F2 |
| **PANTHER PATHWAY** | |  |  |  |
|  | Inflammation mediated by chemokine and cytokine signaling pathway | 7.1 | < 0.001 | PIK3CG, ITGAL, CCL2, CCL8, ITGB2, ITGA4, CCL27, CXCL10, CCRL2, COL14A1, COL7A1, CCL20, CCR5, CCL21, RGS4, CCR2, COL6A3, PLCD4, JAK3, RHOF |
|  | Integrin signalling pathway | 4.6 | 0.014 | PIK3CG, ITGAL, DOCK2, COL14A1, COL7A1, MAPK4, COL6A3, ITGB2, ITGA4, COL8A1, RHOF, COL5A1, FN1 |
|  | Blood coagulation | 2.1 | 0.016 | PLAT, A2M, SERPINF1, F9, PLAU, PROC |
|  | Plasminogen activating cascade | 1.4 | 0.021 | PLAT, SERPINF1, CPB2, PLAU |
| **KEGG PATHWAY** | |  |  |  |
|  | Complement and coagulation cascades | 6.3 | < 0.001 | PLAT, A2M, CFB, C3, C6, F9, SERPING1, C4BPA, C1S, C1QC, PROC, C1QA, C1QB, CFH, C2, CPB2, PLAU, F2R |
|  | PPAR signaling pathway | 3.5 | < 0.001 | CPT2, ACSL1, ACADM, OLR1, APOC3, SLC27A2, DBI, SCP2, CPT1A, PLTP |
|  | Cytokine-cytokine receptor interaction | 6.9 | < 0.001 | CCL2, CXCL2, CXCL9, CCL19, CCL8, CD40, CXCL11, CCL27, IL10, CXCL10, TNFSF13B, CCL20, CCR5, CCL21, IL10RA, CCR2, FAS, XCL1, FIGF, LTB |
|  | Chemokine signaling pathway | 5.6 | 0.001 | PIK3CG, CCL2, CXCL2, CXCL9, CCL19, CCL8, CXCL11, CCL27, CXCL10, DOCK2, CCL20, CCR5, CCL21, CCR2, JAK3, XCL1 |
|  | Systemic lupus erythematosus | 3.8 | 0.001 | C1QA, C1QB, CD86, C3, C6, C1S, CD40, C2, C1QC, FCGR3B, IL10 |
|  | Intestinal immune network for IgA production | 2.4 | 0.004 | CD86, TNFSF13B, MADCAM1, ITGA4, CD40, CCL27, IL10 |
|  | Cell adhesion molecules (CAMs) | 3.8 | 0.007 | VCAM1, ITGAL, CD86, SELL, ICAM3, VCAN, MADCAM1, ITGB2, ITGA4, CD40, PDCD1LG2 |
|  | Glycine, serine and threonine metabolism | 1.7 | 0.014 | CTH, GATM, SDS, MAOA, BHMT |
|  | Prion diseases | 1.7 | 0.022 | C1QA, C1QB, C6, SOD1, C1QC |
|  | Primary immunodeficiency | 1.7 | 0.022 | CD3E, CD79A, JAK3, CD40, BLNK |
|  | Arginine and proline metabolism | 2.1 | 0.023 | ACY1, GATM, P4HA1, MAOA, GLUD1, GLS |
|  | Nitrogen metabolism | 1.4 | 0.033 | CTH, GLUD1, GLS, CA4 |
|  | Toll-like receptor signaling pathway | 2.8 | 0.036 | PIK3CG, CD86, TLR2, CXCL9, CD40, CXCL11, TLR9, CXCL10 |
| **BBID** | |  |  |  |
|  | Chemokine_families | 2.8 | 0.001 | CCL2, CCL20, CCL21, CXCL9, CCL8, CCL19, CXCL11, CXCL10 |
