# Supplementary material for: Renoprotective Mechanism of Remote Ischemic Preconditioning Based on Transcriptomic Analysis in a Porcine Renal Ischemia Reperfusion Injury Model
Source: PLoS One. 2015 Oct 21;10(10):e0141099. doi: 10.1371/journal.pone.0141099 (PMC4619554; doi:10.1371/journal.pone.0141099)
Supplement: S4 Table — (DOCX) [file pone.0141099.s005.docx]

**S4 Table. Cytokines and cytokine receptors with altered gene expression levels.**

|  |  | Ratio* | |
| --- | --- | --- | --- |
| Symbol | Function | rIPCe | rIPCl |
| **Chemokines** |  |  |  |
| CCL2 | Attracts monocytes, memory T cells, and dendritic cells | 1.61 | 2.67 |
| CCL8 | Attracts human leukocytes | 2.05 | 2.99 |
| CCL19 | Attracts dendritic cells, B cells, and memory T cells | 1.49 | 3.01 |
| CCL20 | Attracts lymphocytes and neutrophils | 1.59 | 2.56 |
| CCL21 | Attracts lymphocytes | 1.67 | 2.60 |
| CCL27 | Attracts memory T cells | 0.19 | 0.11 |
| CCR2 | Receptor for CCL2 | 1.88 | 2.22 |
| CCR5 | Receptor for CCL2, 3, and 4 | 1.78 | 3.22 |
| CXCL2 | Attracts polymorphonuclear leukocytes and hematopoietic stem cells | 1.24 | 2.18 |
| CXCL9 | Attracts T cell | 3.80 | 10.15 |
| CXCL10 | Attracts monocytes/macrophages, T cells, NK cells, and dendritic cells | 1.87 | 4.00 |
| CXCL11 | Attracts activated T cells | 4.09 | 8.50 |
| XCL1 | Attracts T cell | 1.32 | 2.14 |
| **Tumor necrosis factor** | | | |
| CD40 | T cell-dependent immunoglobulin class switching, memory B cell development, and germinal center formation | 1.61 | 2.68 |
| FAS | Programmed cell death | 1.54 | 2.66 |
| TNFSF13B | Proliferation and differentiation of B cells | 1.36 | 2.13 |
| LTB | Induces inflammatory response and is involved in normal development of lymphoid tissue | 2.22 | 3.56 |
| **Interleukin** |  |  |  |
| IL-10 | Regulates immune response and inflammation | 1.69 | 2.65 |
| IL10RA | Receptor for interleukin 10 | 1.80 | 3.12 |
| **TGF-β family** |  |  |  |
| TGF-β1 | Regulates immune response and inflammation | 1.32 | 2.00 |

*Ratio: normalized average signal relative values of the rIPCe group versus the control group and the rIPCl group versus the control group. rIPCe, remote ischemic preconditioning with an early time window; rIPCl, remote ischemic preconditioning with a late time window.
